# On/off-switchable anti-neoplastic nanoarchitecture

**DOI:** 10.1038/srep14571

**Published:** 2015-09-29

**Authors:** Hirak K. Patra, Roghayeh Imani, Jaganmohan R. Jangamreddy, Meysam Pazoki, Aleš Iglič, Anthony P. F. Turner, Ashutosh Tiwari

**Affiliations:** 1 Biosensors and Bioelectronics Centre, IFM, Linköping University, 58183, Linköping, Sweden; 2 Integrative Regenerative Medicine Centre, Linköping University, 58185 Linköping, Linköping, Sweden; 3 Division of Cell Biology, Department of Clinical and Experimental Medicine (IKE), Linköping University, 58185 Linköping, Sweden; 4 Laboratory of Biophysics, Faculty of Electrical Engineering, University of Ljubljana, SI-1000 Ljubljana, Slovenia; 5 Laboratory of Clinical Biophysics, Faculty of Health Sciences, University of Ljubljana, SI-1000 Ljubljana, Slovenia; 6 Department of Chemistry, Ångström Laboratory, Uppsala University, Lägerhyddsvägen 1, 75120 Upssala, Sweden; 7 Tekidag AB, Mjärdevi Science Park, Teknikringen 4A, SE 58330 Linköping, Sweden

## Abstract

Throughout the world, there are increasing demands for alternate approaches to advanced cancer therapeutics. Numerous potentially chemotherapeutic compounds are developed every year for clinical trial and some of them are considered as potential drug candidates. Nanotechnology-based approaches have accelerated the discovery process, but the key challenge still remains to develop therapeutically viable and physiologically safe materials suitable for cancer therapy. Here, we report a high turnover, on/off-switchable functionally popping reactive oxygen species (ROS) generator using a smart mesoporous titanium dioxide popcorn (TiO_2_ Pops) nanoarchitecture. The resulting TiO_2_ Pops, unlike TiO_2_ nanoparticles (TiO_2_ NPs), are exceptionally biocompatible with normal cells. Under identical conditions, TiO_2_ Pops show very high photocatalytic activity compared to TiO_2_ NPs. Upon on/off-switchable photo activation, the TiO_2_ Pops can trigger the generation of high-turnover flash ROS and can deliver their potential anticancer effect by enhancing the intracellular ROS level until it crosses the threshold to open the ‘death gate’, thus reducing the survival of cancer cells by at least six times in comparison with TiO_2_ NPs without affecting the normal cells.

Over the past decade, significant effort has been expended^1^,^2^,^3^ to find new approaches for cancer therapy^4^,^5^,^6^,^7^. Compared with chemotherapeutic, surgical, radiological and other conventional therapies, photocatalytic and photodynamic treatments are considered promising alternative strategies for cancer treatment^8^,^9^,^10^,^11^. Nanoparticle-based systems have been intensively investigated in this regard and often emerged as potential key elements in a photocatalytic apparatus for such alternative anticancer therapy^12^,^13^. However, the integration of nanoscale systems into clinical settings has been greatly impeded by the inability to specifically target the tumour cells and by non-specific distribution and damage caused to normal cells^14^,^15^,^16^.

The most promising photocatalytic material is TiO_2,_ following its FDA approval in 1996^17^. It has been useful as a catalyst for photo-degradation of organic compounds and the deactivation of microorganisms. It has been reported that various ROS, such as superoxide (O_2_^•−^), singlet oxygen (^1^O_2_), hydroxyl radical (•OH), hydroperoxyl radical (HO_2_^•^), and hydrogen peroxide (H_2_O_2_), are generated on the TiO_2_ surface and react with organic or inorganic compounds in the gas and liquid phases. Altered redox status and the intrinsic high ROS level in cancer cells due to multiple genetic alterations and high oxidative stress, thus creates a unique opportunity to preferentially eliminate these cells by further induction of ROS insults to open up the ‘death gate’ and deliver the associated therapeutic benefits^18^,^19^.

Recent studies have shown that the geometry of nanoparticles can significantly modulate their photo-catalytic activity^20^. In addition, the photocatalytic activity of TiO_2_ crystals is heavily dependent upon the nanoscale surface structure, including the surface atomic arrangement and coordination, especially when the particle size is reduced to the nanometer scale, leading to a large specific surface area^21^. Based on the backgrounds, we have employed a new type of mesoporous TiO_2_ structure (i.e., TiO_2_ Pops) to explore the effects of nano-architecture on the control of intracellular ROS production. We present a comparative study of the photocatalytic activities of TiO_2_ NPs and Pops in order to clarify the underlying mechanism that regulates the photocatalysis of the nanostructures and to show how such nano-scale engineering switches the intracellular flash ROS in cancer and normal cells. The present communication reports on/off-switchable photon-triggered ROS production, its effect on cancer cells *in vitro* and the comparative biocompatibility of this novel nano-architecture with non-cancerous cells.

The precursor material was synthesised via a sol-gel method and scanning electron microscopy (SEM) images of the calcined mesoporous TiO_2_ Pops, prepared following the solvothermal process, showed controlled morphology and uniform monodisperse sub-micro particles^22^. The precursor beads had smooth surfaces without obvious granular features and a diameter of 600 ± 50 nm (Fig. 1a). After the solvothermal treatment, monodispersed TiO_2_ Pops with a diameter of 500 ± 50 nm and comparatively rough surfaces with surface area up to 100 m^2^/g were produced (Fig. 1b). As illustrated by high magnification SEM images of these TiO_2_ Pops, ~14 nm sized nanocrystals and pores can be observed over the surface (Extended Fig. 1). Mesoporous TiO_2_ Pops were examined using transmission electron microscopy (TEM) to determine their nanoarchitecture, shape and appearance. The morphology of the spherical Pops showed an extensive rough surface composed of a self-organised TiO_2_ nanocrystals that are quite uniform in nature with an average size of 500 nm (Fig. 1c). The crystal facets determined through high-resolution TEM (HRTEM) analysis delineates the lattice fringes of individual nanocrystals with lattice spacing of d = 0.17 nm corresponding to the (105) plane of anatase phase of TiO_2_ (Fig. 1d). The X-ray diffraction(XRD) pattern of these TiO_2_ Pops showed well resolved diffraction peaks (Fig. 1e). The corresponding characteristic 2θ values at 25.00 and 27.50^o^ of the diffraction peaks confirm that the TiO_2_ NPs indeed contain both anatase and rutile phase. TiO_2_ Pops illustrated the characteristic 2θ value at 25.00^o^ of the anatase phase, which indicates that the TiO_2_ Pops consist of highly crystallised anatase without any impurity. The nature of the chemical bonds on both of the samples was analysed using high-energy resolution X-ray photoelectron spectroscopy (XPS). The XPS spectra of Ti 2p, O 1s were fitted into different components and the detailed high-energy resolution analysis is shown in extended Fig. 2. In the Ti 2p spectrum, spin-orbit split doublet Ti 2p_3/2_ and Ti 2p_1/2_ were found to only originate in both samples from the Ti4+ valence state (Ti 2p_3/2_ peak at 458.3 eV). The O 1 s spectra shown three distinct components: i) at 529.5 eV, originating from oxygen atoms in the TiO_2_ oxide lattice, ii) at 531.0 eV, probably related with OH bonds and ii) at 532.5 eV, probably related with H_2_O^23^.

The chemical photocatalytic activity of TiO_2_ NPs and Pops were compared and for the first time we showed a clear and distinct pattern with respect to catalyst amount, substrate concentration and photon exposure time (extended Fig. 3). We have engineered the TiO_2_ flash ROS generator for on-demand photodynamic anti-neoplastics therapy based on the following critical features and dynamicity of particle size, crystal phase and crystallinity on the reactivity. Firstly, the optimal ‘safe’ size is based on numerous examples drawn by Wiesner’s group, which showed clearly that 100 nm is the distinct cut-off size below which atypical surface reactivity arises and causes normal nanoscale toxicity^24^. Secondly, the anatase crystalinity is more prone to interact with the increase in complexity of the membrane and can create membrane leakage more effectively than the rutile form, but does not usually generate ROS^25^; whereas the rutile nanoforms generate ROS and induce general nanotoxicity. Based on these two critical features, we have engineered TiO_2_ Pops having a size greater than 100 nm to avoid normal nanotoxicity and to exploit the anatase phase of the Pops for comparatively selective binding to cancer cells for controlled inducible photodynamic therapy. The intracellular ROS level of prostate cancer cells (PC3) was triggered extensively by photon induced TiO_2_ Pops, but remained unaltered without photon excitation (Fig. 2a). In contrast, the nanoparticle form of the same material (i.e., TiO_2_ NPs) can induce oxidative stress without any photo induction and thus can induce unregulated cytotoxicity in normal embryonic fibroblast cells (MEF), as shown in right panel of Fig. 2a. Upon photoinduction the relative intracellular ROS level increased by 2.5 fold with respect to the untreated control (Fig. 2b). The effective switching of intracellular redox level of the entire population shows δ_1_  δ_2_, i.e., effective high-turnover induction of flash ROS for functionally popping the ‘off-state’ to ‘on-state’ for anti-neoplastic therapy with TiO_2_ pops. The intracellular occurence of such generated ROS is illustrated in Fig. 2c in normal, treated and induced conditions. The effect of the elevated ROS showed as the functional loss of mitochonrial activity.

One of the hallmarks of cancer cells is the presence of dysfunctional and hyperpolarised mitochondria, and this facilitates cancer cell survival by perturbing the release of inter-mitochondrial membrane localised pro-apoptotic factors. The relative mitochondrial reductase activity estimated through a MTT assay using 3-(4,5-dimethylthiazol-2-yl)-2,5-diphenyltetrazolium bromide^26^, showed a reduction of both the mitochondrial function as well as the cell survival rate (Fig. 3a). The controlled manupulation of mitochondrial activity was further estimated in living cells using a Rosamine-based mitochondria-specific dye (MitoTracker Red CMXRos) and showed greater membrane potential-dependent accumulation under control conditions. This implies the depolarisation of the membrane potential when the system was turned from the ‘off-‘ to the ‘on-‘ state (Fig. 3b). The simultaneous fate of the cancer cells was estimated on the basis of changes that occur in the permeability of cell membranes during apoptosis, using PO-PRO (Fig. 3c). Relative simultaneous observations on the same cell population showed that there was a decrese in mirochondrial function and increase in apoptotic population (Fig. 3d). The intracellular space was simultaniously probed using DAPI, Mitotracker Red and DCF-Da to observe the dynamic features of mitochondrial activity with ROS production under live conditions. This clearly showed the trade-off between generating high turnover ROS in ‘on’ condition and the compromised mitochondrial function only in case of Pops (Fig. 3e).

Finally, we considered spatiotemporal control of the ‘on/off’− induction procedure. The normal intracellular ROS level can be greatly impaired by nanoparticles in an uncontrollable fashion because of their nanoscale toxicity, as clearly is shown in Figs 2 and 3. We compared an ‘on/off’− state of the ROS in the presence and absence of photoinduction both in NPs and Pops (Fig. 4a,b). The relative changes were very low in the case of NPs (quantified in our previous set of experiments as δ_2_). However, we successfully established on/off-switching of intracellular ROS generation with our synthesised nanoarchitecture in accordance with our observation as δ_1_ in Fig. 2b. The geometrical exposure profile shows that without any laser we can precisely induce a phototherapeutic effect even over a stretch of 10 cells or around 200 μm. We were able to control cellular ROS expression with a precision of around 100 μm through a ‘pin-hole’ exposure (Fig. 4c). We believe this can be reduced to cellular level through more precise design of the masks.

## Conclusion

Personalised on demand loco-regional therapeutics are emerging as target selective elixir for aggressive diseases like cancer. The most recent thought in this direction is mechanism-based therapy due to plasticity, heterogeneity and clonal evolution in response to the selective pressure imposed by cytotoxic measure and chemotherapeutic agents in tumours. Thus a new paradigm is shifting towards adjustments of treatment regimens by targeting the unusual metabolic flexibility of tumour cells. Reactive oxygen species (ROS) is one such important parameter that regulating redox homeostasis within normal cells and distinctly altered redox status is present in cancer cells. We have exploited this intracellular redox imbalance that provides a biochemical basis for developing novel therapeutic strategies by developing a switchable smart nanoarchitecture that can provide a flash ROS boost. The process is enough to cross the oxidative threshold to selectively kill the cancer cells. This novel ‘fighting fire with fire’ approach offers custom-fit attractive features for developing on/off-switchable functional hand-in-hand activity to design suitable protocols towards the advancement of next generation switchable therapeutics.

## Methods

### Synthesis, characterisation and functional assay of TiO_2_ pops

The mesoporous functionally popping nanoarchitectural TiO_2_ microbreads were synthesised in two steps using the method proposed by Chen *et al.*^22^,^27^,^28^ *Sol-gel procedure for the synthesis of precursor TiO*_*2*_ *sub-micron spheres*: In brief, 28 mM hexadecylamine (HDA from 90% Aldrich), 220 mM DI-water and 0.4 mM KCl were dissolved in ethanol solvent. HDA was used as a structure directing agent and KCl to control the ionic strength in the solution for the synthesis of a smooth and monodispersed morphology. The reaction of the Ti precursor with the oxygen source was completed by adding titanium(IV) isopropoxide (Aldrich, 97%, 70 mM) under vigorous stirring at room temperature. The solution was left static for 18 h under a fume hood and then centrifuged and dried at room temperature. A solvothermal method was used for surface treatment of the resulting TiO_2_ sub-micro particles. They were dispersed in a mixture of ethanol/DI-water (2:1, v/v) which contained 0.55 M ammonia solution (Merck, 25%). The solution was transferred into a teflon-lined autoclave and placed in a furnace for 18 h at 170 °C. Finally, the suspension was centrifuged and the mesoporous particles were dried in air at room temperature.

The particle morphology was observed using a Hitachi S4700 field-emission scanning electron microscope (SEM, Hitachi-S4160). Transmission electron microscopy (TEM) was performed using a Jeol JEM-2100 operating at 200 kV to determine the overall shape and appearance of the mesoporous TiO_2_ Pops. High-resolution TEM (HRTEM) analysis was employed to determine the crystal facets. Lattice fringes of individual nanocrystals were imaged with HRTEM. The crystal structure properties of the TiO_2_ nanostructures were obtained from hard X-ray low-angle reflectivity measurements, using a Philips PW1710 powder diffractometer with a copper anode source (Cu K*α*, lambda = 1.54 Å), operating at 0.8 kW and with an accuracy of 0.015° 2-theta. For surface chemistry charaterisation, X-ray photoelectron spectroscopy (XPS or ESCA) analyses were carried out on a PHI-TFA XPS spectrometer manufactured by Physical Electronics Inc. The analysed area was 0.4 mm in diameter and the depth was about 3–5 nm. Sample surfaces were excited by X-ray radiation from a monochromatic Al source at photon energy of 1486.60 eV. The high-energy resolution spectra were acquired with an energy analyser operating at a resolution of about 0.60 eV and pass energy of 29 eV.

In order to study ROS generation, photocatalysis experiments were performed by adding 5 mg TiO_2_ particles to 5 mL ethanol, and the suspension was sonicated for ten min. Then, 1 × 10^−3^ g/L 1,5-diphenylcarbazide (DPCI) was added to the suspension, and the mixture was stirred magnetically for fifteen min in the dark. The beaker containing the suspension was placed under a 4 W UV-handheld lamp and irradiated at 365 nm. The distance between the beaker and the light source was 5 cm, the reaction temperature was maintained at 25 °C. After irradiation, carbon tetrachloride was added to solution for extraction of 1,5-diphenylcarbazone (DPCO). After the final extraction, UV/vis absorption spectra of the solutions were recorded.

### On/off-switchable photocatalysis

For the photo- activation and switching to the on- state of the therapeutic procedure, we operated under GMP conditions using a Labino BigBeam light source enabled with LED technology for a smooth uniform beam (9 × 365 nm UV-A LED’s) and narrow spectra with a peak of 365 nm. The exposure is completely (100%) free from harmful UV-B and the diodes emit almost no other wavelength with an output power of 3.5 mw/cm^2^ at 38 cm, with beam diameter 250 mm. The dose was optimised to an exposure of 180 sec. The dose was non-toxic to the normal embryonic fibroblast cells (MEF). There was no production of ROS with this photon exposure.

### Intracellular ROS estimation

Both cancer (PC3) and normal cells were cultured up to 70% confluency and around 10^6^ cells in their respective medium and then treated with TiO_2_ Pops and NPs, with a final concentration of 50 μg/mL, and incubated for 12 h. The cells were then washed and incubated with 5 μM 2′,7′-dichlorofluorescin diacetate (DCF-Da) in PBS for 30 min at 37 °C. The cells were then photo induced as described in the supplementary section. The entire population was shifted to produce high amount of ROS after TiO_2_ Pops mediated photoinduction in PC3 cells.

### Flow cytometric measurement of mitochondrial activity and apoptosis

Cells plated and treated as mentioned for ROS estimation were then stained with 100 nM Mitotracker Red CMXROS (Life Technologies Inc.) for 45 min before trypsinisation. After collecting the trypsinised and resuspended cells in the medium the cells were centrifuged for 5 min at 300 g, washed with PBS and resuspended in 500 μL of PBS and incubated with Po-Pro dye (Life Technologies Inc.) for 30 min on ice as per the manufacturer’s instructions^29^. The cells were then subjected to flow cytometry using FL4 and FL9 filters for Mitotracker Red and Po-Pro respectively (Gallios) and analysed using FlowJo software (vX.0.7).

### Confocal microscopy

The cells treated or untreated as mentioned were fixed using 4% paraformaldehyde and imaged using Zeiss LSC microscopy as described previously in detail^29^.

## Additional Information

**How to cite this article**: Patra, H. K. *et al.* On/off-switchable anti-neoplastic nanoarchitecture. *Sci. Rep.* **5**, 14571; doi: 10.1038/srep14571 (2015).

## Supplementary Material

Supplementary Information

## Figures and Tables

**Figure 1:**
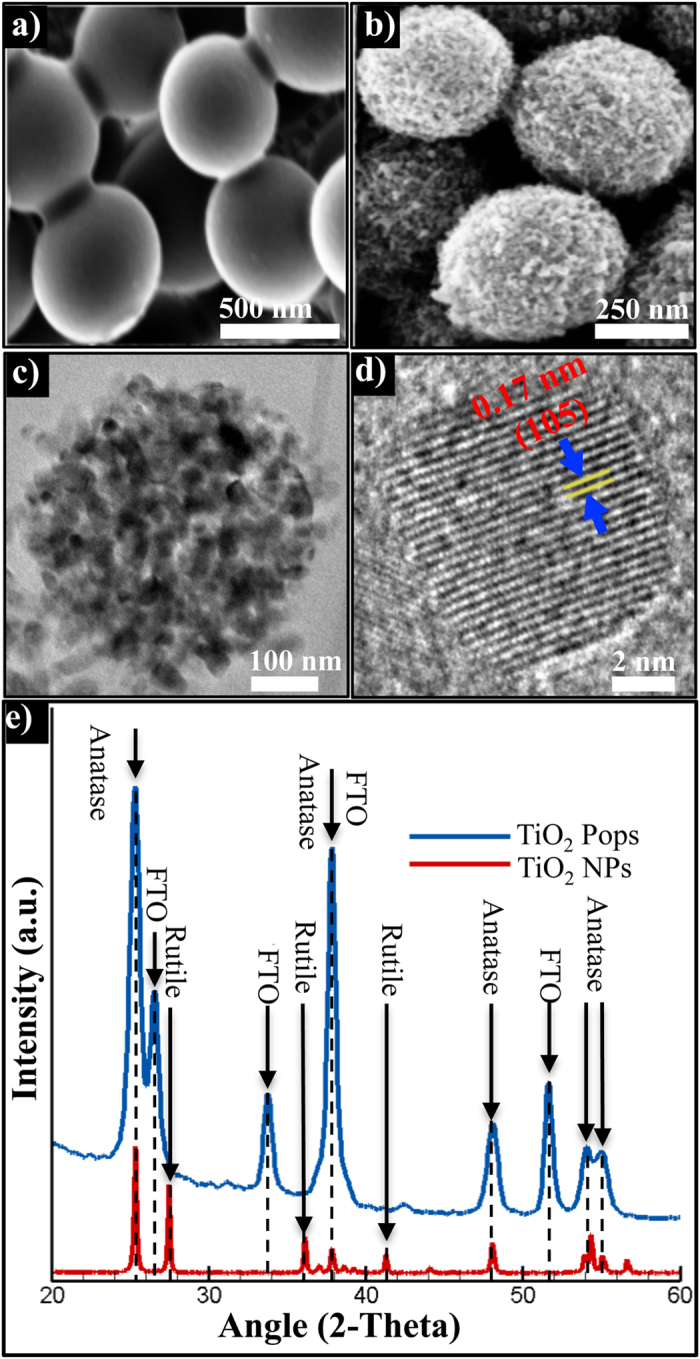
SEM images of mesoporous TiO_2_ Pops, (**a**) micro-particles before solvothermal treatment, (**b**) mesoporous Pops after solvothermal treatment. (**c**) TEM image of solvothermally engineered mesoporous TiO_2_ nano-crystalls. (**d**) Pops nanocrystals with lattice spacing of d = 0.17 nm corresponding to the (105) plane of anatase phase of TiO_2_ in HRTEM. (**e**) Comparative X-ray diffraction patterns of mesoporous TiO_2_ Pops and NPs.

**Figure 2:**
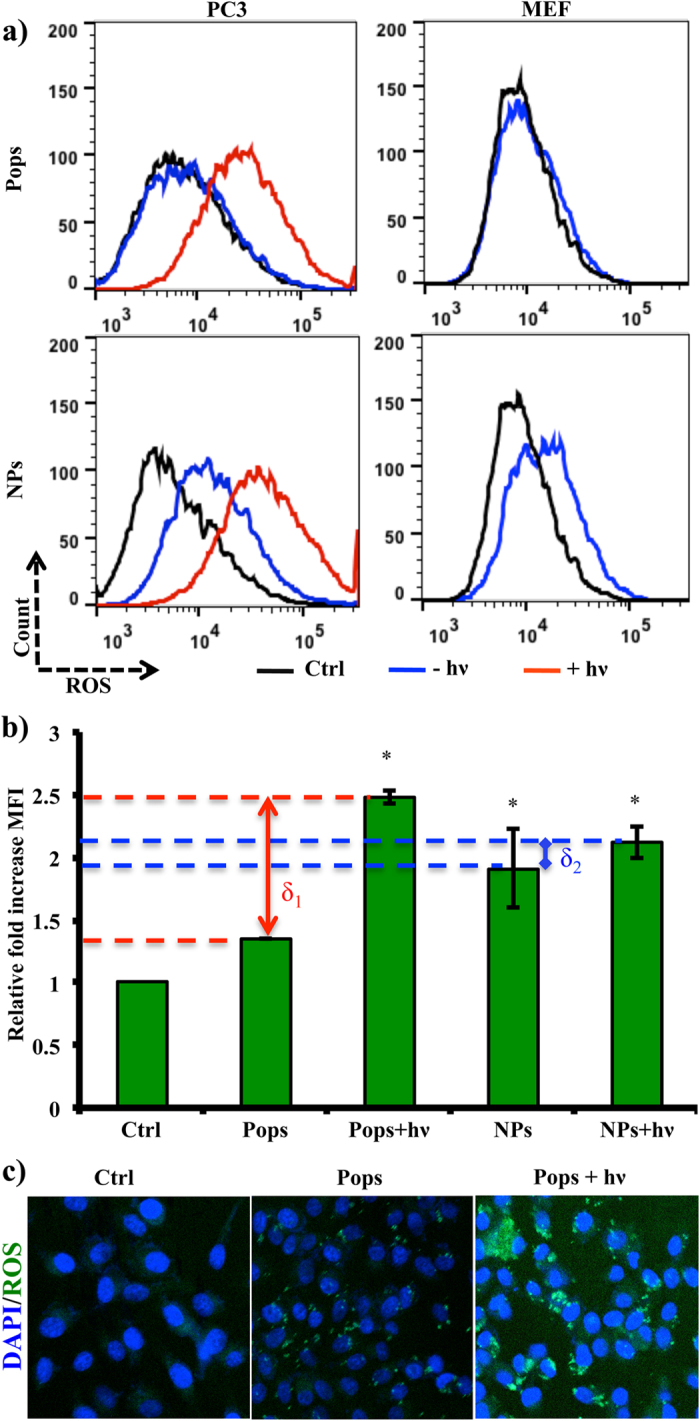
*In vitro* comparative intracellular ROS generation profile in cancer and normal cells. Both cancer (PC3) and normal cells were cultured upto 70% confluency and around 10^6^ cells in their respective medium and then treated with Pops and NPs with 50 μg/mL and incubated for 12 h. The cells were then washed and incubated with 5 μM 2′,7′-dichlorofluorescin diacetate (DCF-Da) in PBS for 30 min at 37 °C. The cells were photo induced as described in the supplementary section. The entire population was shifted to produce a high amount of ROS after TiO_2_ Pops-mediated photoinduction in the PC3 cells (**a**). Notably, only Pops did not induce any ROS in PC3 and normal MEF cells (right panel). In contrast, NPs can itself induced ROS without any photoinduction (lower panel). The ROS expression level of the relative population in presence and absence of photon (**b**). Significant changes observed in nanocrystalline architure compared to NPs (**b**). Intracellular ROS localisation and amount under control, treated and induced conditions (**c**).

**Figure 3:**
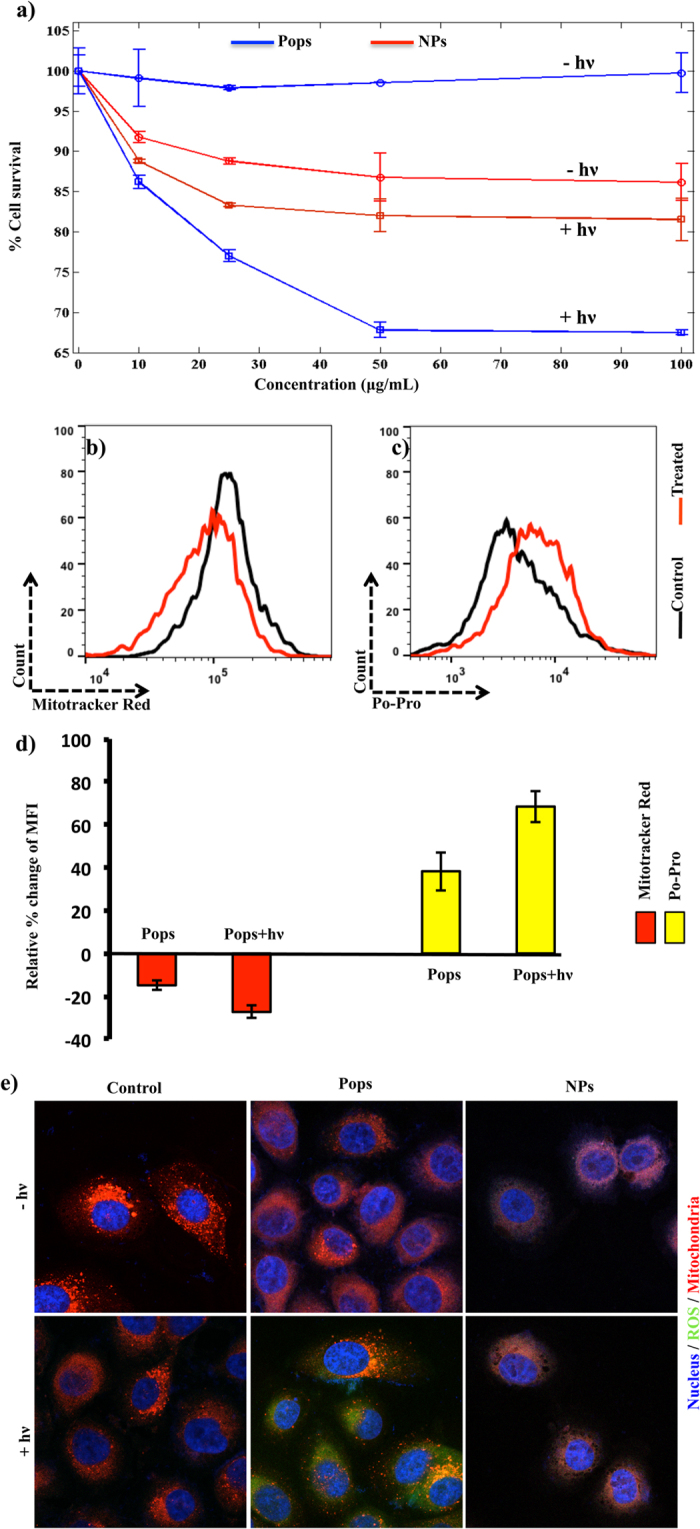
Effect of TiO_2_ nanoarchitecture: Concentration dependent estimation of the % cell survival by measuring the functional activity of the mitochondrial dehydrogenase within the live cells in ‘on-state’ and ‘off-state’ photo indduction (a). Flow cytometric measurement of mitochondrial membrane potential (Mitotracker red) and simultaneous determination of the onset of apoptotic population (**b**–**c**). The inverse correlation of the mitochondrial membrane potential and apoptosis in on- state of the therapy (**d**). The intracellular localisation and the dynamics of mitochondrial function and ROS in presence and absence of photoinduction and compared between Pops and NPs (**e**) in PC3 cells.

**Figure 4:**
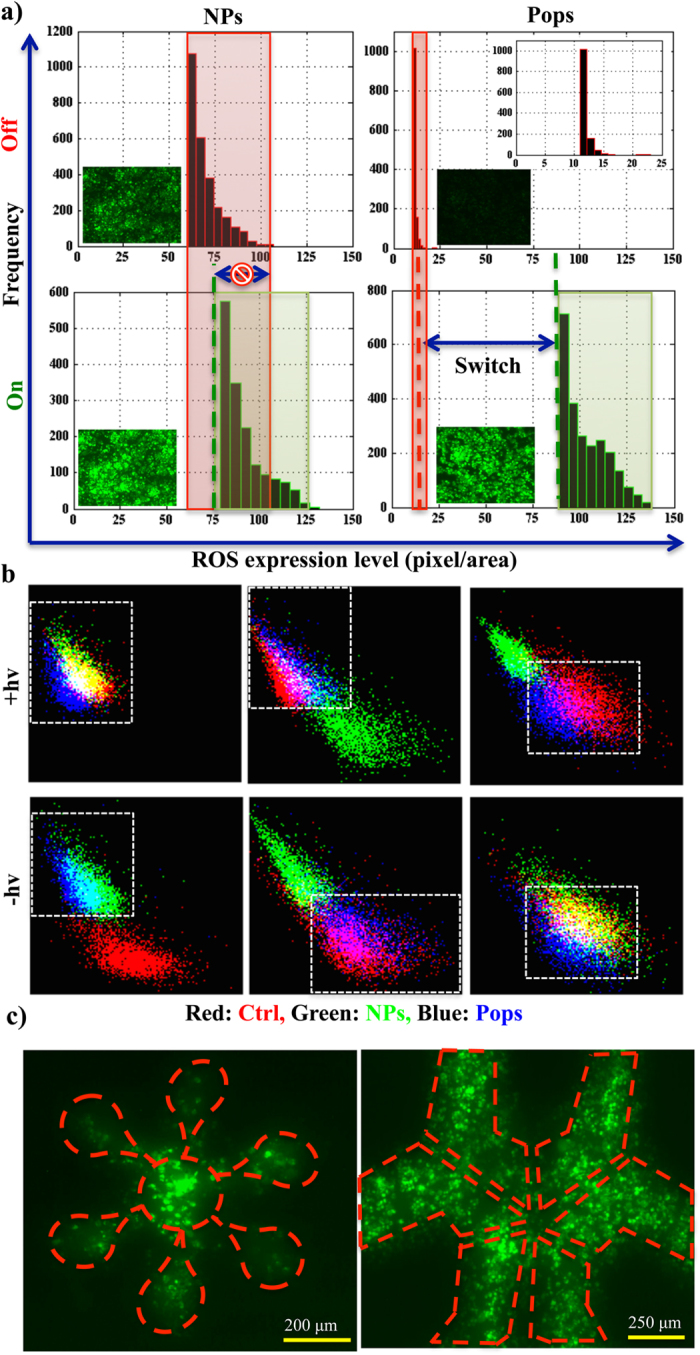
On/off-switchable anti-neoplastic therapeutics and precision. Comparative three dimensional densitometric ROS level in the off and on state for fluorescence microscopic images (inset, 10X) in the presence and absence of photoinduction for nanoparticle (left panel) and Pops (right panel) treated PC3 cells (**a**). The simultaneous expression of ROS (y-axis) and mitochondrial activity (x-axis) in ‘on/off-state’ (upper/lower panel) in normal (MEF, right panel), transformed (HEK, left panel) and cancer (PC3, middle panel) cells (**b**). The geometrical and spatial precision for loco-regional theraputic activation (**c**).

## References

[b1] Tammela T. *et al.* Photodynamic ablation of lymphatic vessels and intralymphatic cancer cells prevents metastasis. Science Trans. Med. 3, 69ra11: 1–8 (2011). 10.1126/scitranslmed.3001699 21307301

[b2] Kennedy J. E. High-intensity focused ultrasound in the treatment of solid tumours. Nat. Rev. Cancer 5, 321–327 (2005). 15776004 10.1038/nrc1591

[b3] Lukianova-Hleb E. Y. *et al.* On-demand intracellular amplification of chemoradiation with cancer-specific plasmonic nanobubbles. Nat. Medicine 20, 778–784 (2014). 10.1038/nm.3484 PMC4271542 24880615

[b4] Millar A. W., & Lynch K. P. Rethinking clinical trials for cytostatic drugs. Nat. Rev. Cancer 3, 540–545 (2003). 12835674 10.1038/nrc1124

[b5] Gorski D. H. Integrative oncology: really the best of both worlds? Nat. Rev. Cancer 14, 692–700 (2014). 10.1038/nrc3822 25230880

[b6] Tiwari A., Patra H. K. & Choi J.-W. (Eds.), In Advanced Theranostic Materials. Wiley-Scrivener, Beverly, USA, 2015.

[b7] Patra H. K. *et al.* MRI-visual order-disorder micellar nanostructures for smart cancer theranostics. Adv. Healthcare Mater. 3, 526–535 (2014). 10.1002/adhm.201300225 23983185

[b8] Schroeder A. *et al.* Treating metastatic cancer with nanotechnology. Nat. Rev. Cancer 12, 39–50 (2011). 22193407 10.1038/nrc3180

[b9] Collins I. & Workman P. New approaches to molecular cancer therapeutics. Nat. Chem Biol. 2, 689–700 (2006). 17108987 10.1038/nchembio840

[b10] Essmann F. & Schulze Osthoff K. Translational approaches targeting the p53 pathway for anti-cancer therapy. Br. J. Pharmacol. 165, 328–344 (2012). 21718309 10.1111/j.1476-5381.2011.01570.x PMC3268188

[b11] Rehman F. L., Lord C. J. & Ashworth A. Synthetic lethal approaches to breast cancer therapy. Nat. Rev. Clin. Oncol. 7, 718–724 (2010). 20956981 10.1038/nrclinonc.2010.172

[b12] Pasparakis G., Manouras T., Vamvakaki M. & Argitis P. Harnessing photochemical internalization with dual degradable nanoparticles for combinatorial photo–chemotherapy. Nat. Com. 5, 3623, 1–9 (2014). 10.1038/ncomms4623 PMC3988806 24710504

[b13] Bourzac K. Nanotechnology: carrying drugs. Nature 491, S58–S60(2012). 23320289 10.1038/491s58a

[b14] DeLoid G. *et al.* Estimating the effective density of engineered nanomaterials for *in vitro* dosimetry. Nat. Com. 5, 3514, 1–10 (2014). 10.1038/ncomms4514 PMC4038248 24675174

[b15] Auffan M. *et al.* Towards a definition of inorganic nanoparticles from an environmental, health and safety perspective. Nat. Nano. 4, 634–641 (2009). 10.1038/nnano.2009.242 19809453

[b16] Wright J. Nanotechnology: Deliver on a promise. Nature 509, S58–S59(2014). 24870822 10.1038/509S58a

[b17] Sadrieh N. Overview of CDER experience with nanotechnology-related drugs. In: Slides for the August 9, 2012 Meeting of the Advisory Committee for Pharmaceutical Science and Clinical Pharmacology. 2012. http://www.fda.gov/downloads/AdvisoryCommittees/CommitteesMeetingMaterials/Drugs/AdvisoryCommitteeforPharmaceuticalScienceandClinicalPharmacology/UCM315773.pdf. Accessed 14 February 2015.

[b18] Trachootham D., Alexandre J. & Huang P. Targeting cancer cells by ROS-mediated mechanisms: a radical therapeutic approach? Nat. Rev. Drug Dis. 8, 579–591 (2009). 10.1038/nrd2803 19478820

[b19] Raj L. *et al.* Selective killing of cancer cells by a small molecule targeting the stress response to ROS. Nature 475, 231–234 (2011). 21753854 10.1038/nature10167 PMC3316487

[b20] Li Y. & Liu Z. Particle size, shape and activity for photocatalysis on titania anatase nanoparticles in aqueous surroundings. J. Am. Chem. Soc. 133, 15743–15752 (2011). 21879719 10.1021/ja206153v

[b21] Kisch H. Semiconductor Photocatalysis—Mechanistic and Synthetic Aspects. Angew. Chem. Intl. Ed. 52, 812–847 (2013). 10.1002/anie.201201200 23212748

[b22] Chen D. *et al.* Synthesis of monodisperse mesoporous titania beads with controllable diameter, high surface areas, and variable pore diameters (14− 23 nm). J. Am. Chem. Soc. 132, 4438–4444 (2010). 20201515 10.1021/ja100040p

[b23] Song Z., Hrbek J. & Osgood R. Formation of TiO_2_ nanoparticles by reactive-layer-assisted deposition and characterization by XPS and STM. Nano Let. 5, 1327–1332 (2005). 16178232 10.1021/nl0505703

[b24] Maynard A. D. Don’t define nanomaterials. Nature 475, 31–31 (2011). 21734686 10.1038/475031a

[b25] Braydich-Stolle L. *et al.* Crystal structure mediates mode of cell death in TiO_2_ nanotoxicity. J. Nanopart. Res. 11, 1361–1374 (2009).

[b26] Ebada S., Edrada R., Lin W. & Proksch P. Methods for isolation, purification and structural elucidation of bioactive secondary metabolites from marine invertebrates. Nat. Prot. 3, 1820–1831 (2008). 10.1038/nprot.2008.182 18989260

[b27] Imani R. *et al.* Band edge engineering of TiO_2_@DNA nanohybrids and implications for capacitive energy storage devices. Nanoscale 7, 10438–10448 (2015). 26001096 10.1039/c5nr02533h

[b28] Pazoki M. *et al.* Mesoporous TiO_2_ microbead electrodes for solid state dye sensitized solar cells. RSC Adv. 4, 50295–50300 (2014).

[b29] Jangamreddy J. *et al.* Salinomycin induces activation of autophagy, mitophagy and affects mitochondrial polarity: differences between primary and cancer cells. Bioch. Biophys. Acta (BBA)-Molecular Cell Research. 1833, 2057–2069 (2013). 10.1016/j.bbamcr.2013.04.011 23639289

