# Supplementary material for: On/off-switchable anti-neoplastic nanoarchitecture
Source: Sci Rep. 2015 Sep 29;5:14571. doi: 10.1038/srep14571 (PMC4586894; doi:10.1038/srep14571)
Supplement: Supplementary Information [file srep14571-s1.doc]

**Supporting Information**

**On/off-switchable anti-neoplastic nanoarchitecture**

Hirak K. Patra^a,b,c,#^, Roghayeh Imani^a,d,e#^, Jaganmohan R. Jangamreddy^c^, Meysam Pazoki^e^, Aleš Iglič^d,*^, Anthony P. F. Turner^a^ and Ashutosh Tiwari^a,g,*^

^a^ Biosensors and Bioelectronics Centre, IFM, Linköping University, 58183, Linköping, Sweden

^b^ Integrative Regenerative Medicine Centre, Linköping University, 58185 Linköping, Linköping, Sweden

^c^ Division of Cell Biology, Department of Clinical and Experimental Medicine (IKE), Linköping University, 58185 Linköping, Sweden

^d^ Biophysics Laboratory, Faculty of Electrical Engineering, University of Ljubljana, SI-1000 Ljubljana, Slovenia

*^e^* *Laboratory  of  Clinical Biophysics, Faculty of  Health  Sciences, University of  Ljubljana, SI-1000 Ljubljana, Slovenia*

*^f^ Department of Chemistry, Ångström Laboratory, Uppsala University, Lägerhyddsvägen 1751 20 Upssala, Sweden*

*^g^ Tekidag AB, Mjärdevi Science Park, Teknikringen 4A, SE 583 30 Linköping, Sweden*

*^#^Authors contributed equally and names arranged in alphabetical order.*

**Corresponding authors.* E-mail: [ales.iglic@fe.uni-lj.si](mailto:ales.iglic@fe.uni-lj.si), Tel: (+386) 1476 88 25, Fax: (+386) 1476 88 50 (A. Iglič); E-mail: [ashutosh.tiwari@liu.se](mailto:ashutosh.tiwari@liu.se), Tel: (+46) 13 282395, Fax: (+46) 13 137568 (A. Tiwari).

**Extended Figures:**


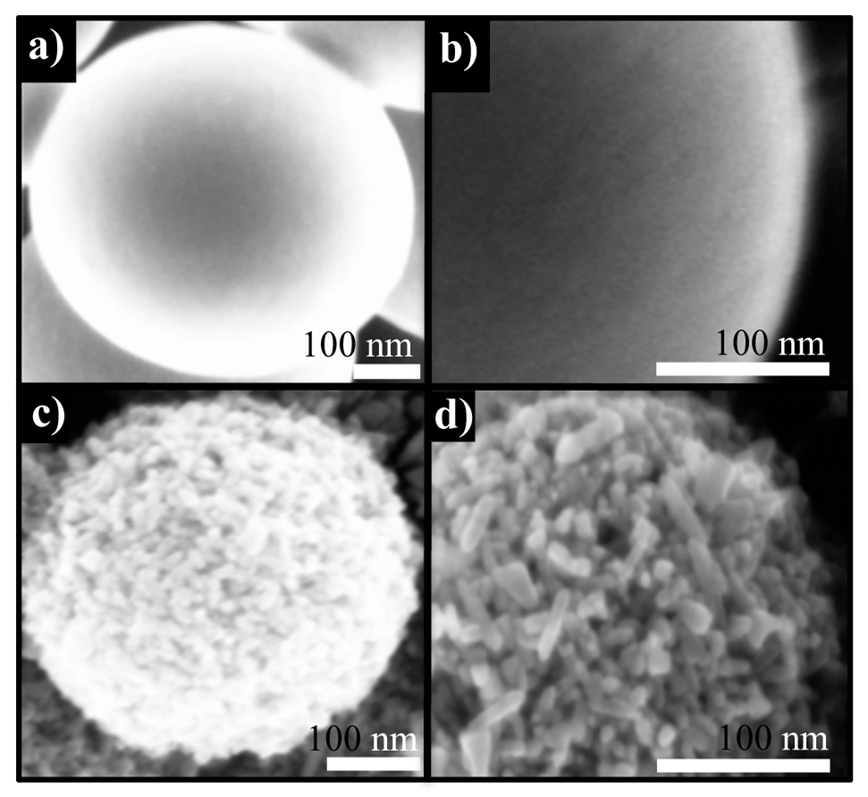


Extended Figure 1: High resolution SEM image before (a,b) and after (c,d) nano engineering of the TiO_2_ particle. This illustrates and confirms the formation of ~14 nm nanocrystals and pores over the surface of the particles (d) and emerging with on/off switchable photocatalytic functions.


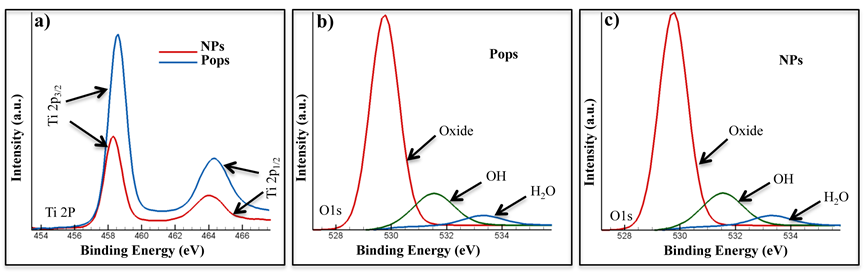


Extended Figure 2: Chemical bonding (High energy XPS resolution spectra) (a) Ti 2p XPS obtained on the surface of the mesoporous TiO_2_ Pops and NPs, (b) O 1s, XPS high energy resolution spectra obtained on the surface of the mesoporous TiO_2_ Pops and (c) O 1s, XPS high energy resolution spectra obtained on the surface of the TiO_2_ NPs.


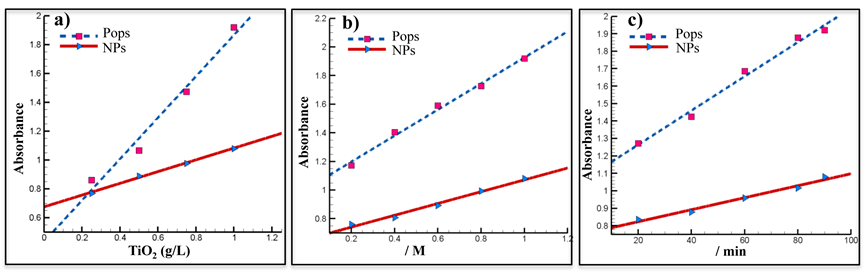


Extended Figure 3: Chemical Photocatalysis: Absorption change of DPCO extract with a) changing the catalyst amount, DPCI = 1 mg/L, irradiation time = 90 min, b) changing the DPCI amount, catalyst = 1 g/L, irradiation time = 90 min and c) changing the irradiation time, catalyst = 1 g/L, DPCI = 1 mg/L.


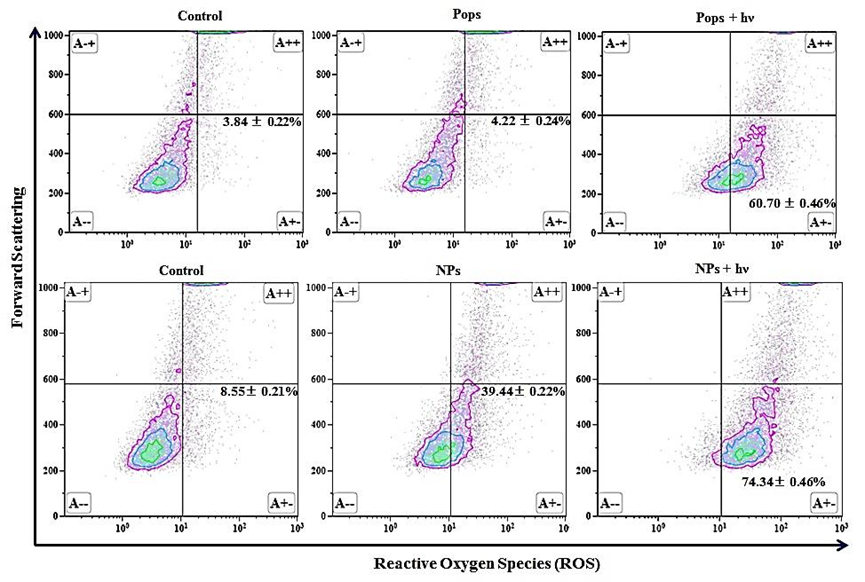


Extended Figure 4: The flow cytometry study using NPs and Pops in presence and absence of hν on cancer cells (PC3).

**Redox balance in normal and cancer cells:**

The intracellular redox condition is highly regulated and maintained a homeostasis of ROS that plays a crucial role as a second messenger in normal metabolic function, survivality and proliferation of the normal cells. ROS generated through various intracellular pathways are regulated by antioxidant procedures such as antioxidants or enzymatic scavengers. But in cancer cells, there are increased level of ROS and an altered redox status due to oncogene activation, higher metabolic rate, faster proliferation rate and lower antioxidant capacity. Ironically, it has also been proved that existing chemo and radiotherapeutic procedures also elevate the ROS level in treated cancers to trigger the cell death. In the present project we are proposing to use the altered redox state of normal and cancer cells to selectively induce the on/off switchable model using biocompatible TiO_2_ Pops as an alternative anti-neoplastic therapeutic approach through selective ‘ROScution’ (**Scheme 1**).


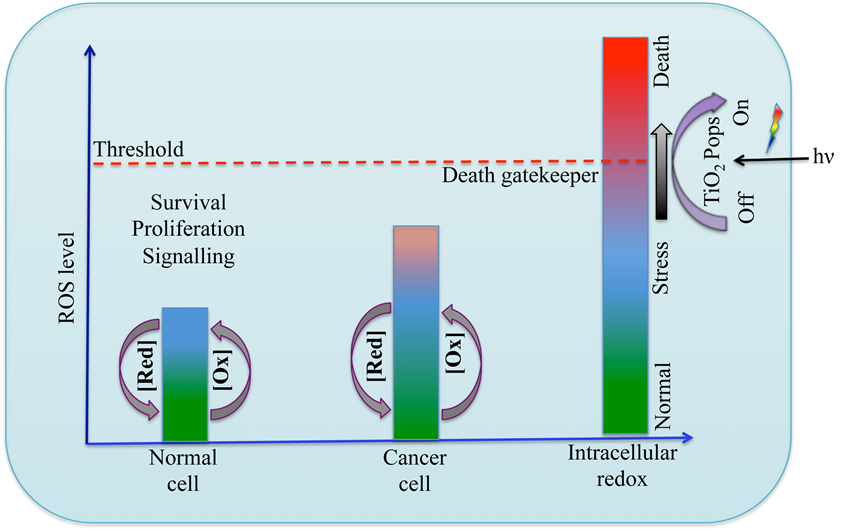


Supplementary Scheme 1: The concept of on/off-switchable anti-neoplastic ‘ROScution’.


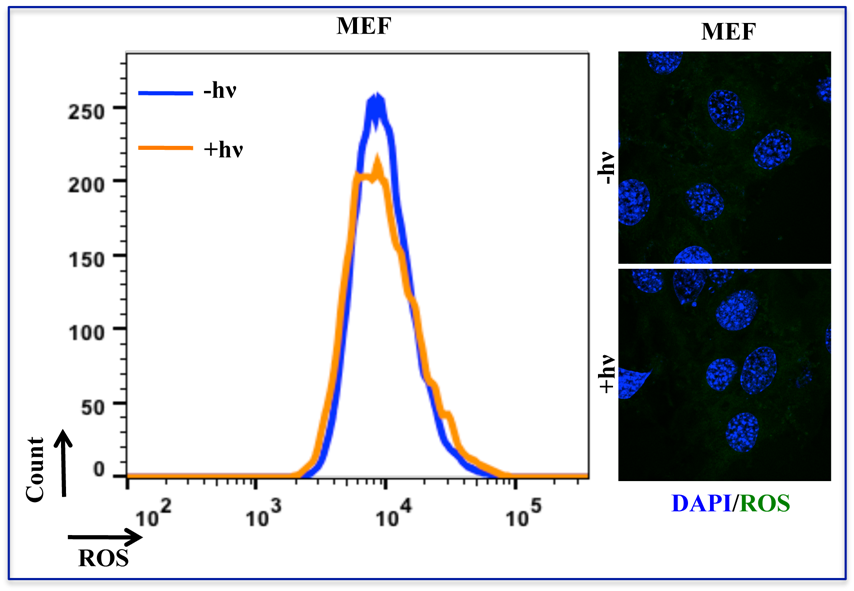


Supplementary Figure 1: Photon dose optimisation with normal cell (MEF) for the functionally popping TiO_2_ nanoarchitecture in anticancer therapy. In presence and absence of the photon, the intracellular ROS level remains same and there is no shift of cell population due to expression of high ROS as we observed in Figure 2a. The right panel describes the intracellular ROS generation with and without exposure of hv applied in this study.

**Mathematical analysis using MATLAB: Quantitative statistical data for on/off- switching in flow cytometry and confocal imaging:**

The LMD files were transformed using FlowJo into csv file. The following MATLAB scripts have been developed to analyse the on/off-switching by simultaneous monitoring the intracellular ROS production and mitochondrial functions. The two dimensional (2D) data (ROS, mt-CMXROS) has been converted into a 2D grid where the matrix elements are indicating events occurrence density. The different treatments (NPs and Pops) and the untreated control for each individual cell type (MEF: normal; HEK: transformed; cancer: PC3) experimental matrices were aligned into three independent colour planes (RGB). The pure colours are for the population of independent nature (red: control; green: NPs; blue: Pops) and the superposed colour representing in the grid points having overlapping population (where different populations are showing similar trait in terms of ROS generation and respective consequences in mitochondrial function). Each such set of results is represented as RGB image. The image rotation is adjusted in such a way that XY-axes are representing the conventional XY co-ordinates and not the image axes. The scaling of the image is represented along a normalised log scale.

Script 1: Mother script

for i=1:3

fhek=['Input folder HEK' num2str(i) '/'];

fmef=['Input folder MEF' num2str(i) '/'];

fpc3=['Input folder PC3' num2str(i) '/'];

[ah,bh]=fxtract(fhek,'csv')

[am,bm]=fxtract(fmef,'csv')

[ap,bp]=fxtract(fpc3,'csv')

zm1=am{1};zm2=am{2};zm3=am{3};

zp1=ap{1};zp2=ap{2};zp3=ap{3};

zh1=ah{1};zh2=ah{2};zh3=ah{3};

zm1n=am{4};zm2n=am{5};zm3n=am{6};

zp1n=ap{4};zp2n=ap{5};zp3n=ap{6};

zh1n=ah{4};zh2n=ah{5};zh3n=ah{6};

subplot(3,2,1);

zz=hirak_facs_stage2(zh1,zh2,zh3,200,200)

subplot(3,2,2);

zz=hirak_facs_stage2(zh1n,zh2n,zh3n,200,200)

subplot(3,2,3);

zz=hirak_facs_stage2(zp1,zp2,zp3,200,200)

subplot(3,2,4);

zz=hirak_facs_stage2(zp1n,zp2n,zp3n,200,200)

subplot(3,2,5);

zz=hirak_facs_stage2(zm1,zm2,zm3,200,200)

subplot(3,2,6);

zz=hirak_facs_stage2(zm1n,zm2n,zm3n,200,200)

figure

end

Script 2: Sub program (fxtract)

function [zz,fname]=fxtract(path,extension)

fname={};zz={};

z=dir([path '*.' extension ]);

l=length(z);

for i=1:l

fname{i}=z(i,1).name;

zz{i}=importdata([path fname{i}]);

end

Script 3: Sub program (hirak_facs_stage2)

function zz=hirak_facs_stage2(z1,z2,z3,m,n)

% z1 ,z2,z3 are images in three planes

x1=z1(:,3);y1=z1(:,4);

x2=z2(:,3);y2=z2(:,4);

x3=z3(:,3);y3=z3(:,4);

zz=hirak_grid_color(x1,y1,x2,y2,x3,y3,m,n);

Script 4: Sub program (hirak_grid_color)

function zz=hirak_grid_color(x1,y1,x2,y2,x3,y3,m,n);

z1=j_grid(-log(y1),log(x1),m,n);

z2=j_grid(-log(y2),log(x2),m,n);

z3=j_grid(-log(y3),log(x3),m,n);

zz(:,:,1)=z1;

zz(:,:,2)=z2;

zz(:,:,3)=z3;

zz=zz/max(max(max(zz)));

zz=255*zz;

zz=uint8(zz);

imshow(zz);

Script 5: Sub program (j_grid_color)

function zz=j_grid_color(x1,y1,x2,y2,m,n);

z1=j_grid(-log(y1),log(x1),m,n);

z2=j_grid(-log(y2),log(x2),m,n);

zz(:,:,1)=z1;

zz(:,:,2)=z2;

zz(:,:,3)=zeros(size(z1));

zz=zz/max(max(max(zz)));

zz=255*zz;

zz=uint8(zz);

imshow(zz)

Script 6: Sub program (j_grid)

function z=j_grid(x,y,m,n);

% m & n are sizes of the matrix

z=zeros(m,n);

xl=linspace(min(x),max(x),m);

yl=linspace(min(y),max(y),n);

for i=1:m- 1;

for j=1:n-1

kk=find ( (x>xl(i) & x<xl(i+1))&(y>yl(j) & y<yl(j+1)));

if isempty(kk)~=1

z(i,j)=z(i,j)+1;

end

end

end

The quantitative script for densitometric ROS

function [idx,g,ga,grain_areas]=imaana1(imname)

i1=rgb2gray(imname);

i2=im2bw(i1,graythresh(i1));

%%%%%%%%%%%%%%%%%%%%%%%%%%%%%%%%%%%%%%%%%%%%%%

% Find the number of distinct objects

% the algorithm may fail if the objects are overlapping

bw = bwareaopen(i2, 50);

cc = bwconncomp(bw, 8);

idx=cc.NumObjects;

g={};ga={};

labeled = labelmatrix(cc);

RGB_label = label2rgb(labeled, @spring, 'c', 'shuffle');

subplot(224);imshow(RGB_label);title('Labeled Object Distribution');

subplot(221);imshow(i1);subplot(222);imshow(

graindata = regionprops(cc, 'basic');

grain_areas = [graindata.Area];

for i=1:idx;

% showing objects consecutively

grain = false(size(bw)); % making a null (black image of size bw)

grain(cc.PixelIdxList{i}) = true; % whitening the object pixels

ga{i}=graindata(i).Area;

grain=uint8(255*grain);

grain(find(grain==255))=i1(find(grain==255));

g{i}=imcrop(grain,graindata(i).BoundingBox);

%figure, subplot(121);imshow(g{i});subplot(122);plot(sum(double(g{i})));axis('square');% showing the image

end

GAA=[];

for i=1:idx

GAA=[GAA; sum(sum(double(g{i})))/ga{i}];

end

subplot(122);hist(GAA);xlabel('Pixel. Area^{-1}');ylabel('Counts');

Subscript: imxtract

function [fname,cw]=imxtract(path,extension)

fname={};cw={};

z=dir([path '*.' extension ]);

%ss=[];im={};bw=[];th=[];kk=[];

frac=[];

k=length(z);

count=1;

for i=1:k

fname{i}=z(i,1).name;

cw{i}=imread([path fname{i}]);

end

**Three step assay procedure for intracellular ROS estimation:**


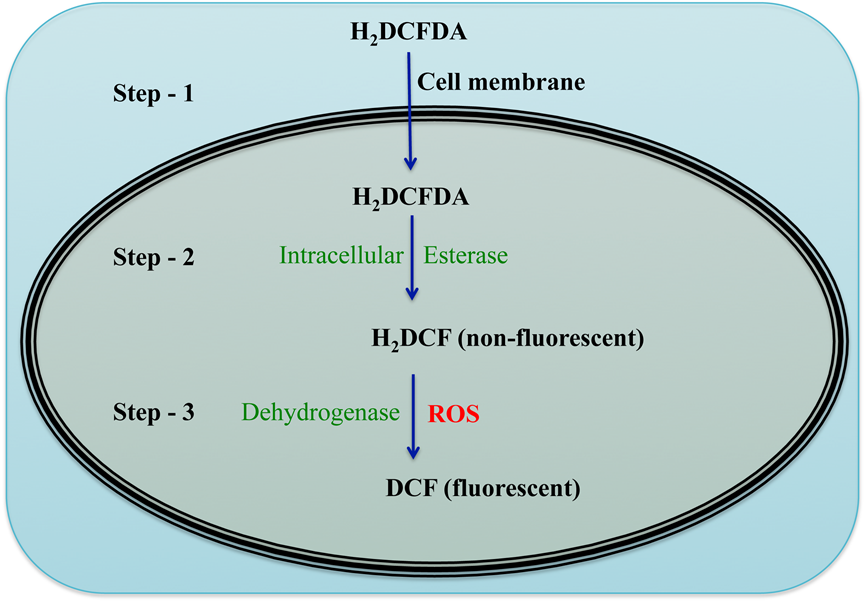


Supplementary Figure 2: A three-step assay system for the estimation of the intracellular ROS within live cells using the fluorescence dye 2’,7’– dichlorofluorescin diacetate (DCFDA). After passing the cell membrane the DCFDA is deacetylated by the intracellular esterase to non-fluorescent compound and that can be oxidized by ROS into 2’,7’–dichlorofluorescin (DCF). We have quantitatively estimated this highly fluorescent DCF in flow cytometry and confocal microscopy.
